# Social Determinants of Voice Outcomes: The Configurational Analysis of the Effects of LMX and Peer Relationships

**DOI:** 10.3390/bs12060197

**Published:** 2022-06-19

**Authors:** Jeeyoung Kim, Ah Jung Kim, Myung-Ho Chung

**Affiliations:** 1 Ewha School of Business, Ewha Womans University, 52-Ewhayeodae-gil, Seodaemun-gu, Seoul 03760, Korea; jeeyoungkim1@ewha.ac.kr; 2 Tippie College of Business, University of Iowa, 21 E Market St, Iowa City, IA 52242, USA; ahjung-kim@uiowa.edu

**Keywords:** voice behavior, individual influence, LMX, peer relationships, social networks

## Abstract

From the perspective of social relationships, this study extends the understanding of employee voice by examining voice outcomes, especially a voicer’s influence in their work team. In particular, we explore how two different social relationships, LMX and peer relationship, separately and jointly affect the ‘voice-influence’ relationship. Drawing on social network theory, we propose that higher LMX and central positions in peer networks (i.e., centrality in the friendship network) strengthen the positive impact of voice on individual influence. From a sample of 128 employees from three firms in South Korea, we found that two types of voice (promotive and prohibitive) are positively related with individual influence. This study also found that LMX strengthened the positive effect of promotive voice on a voicer’s influence. Moreover, LMX and peer relationship jointly affect the voice-influence relationship as follows: (1) a voicer with a high LMX-high centrality (in the peer network) is most influential within their team, (2) as for a low LMX-high centrality member, speaking up rather decreases individual influence. These results suggest that voice outcome is not unilateral. Rather, whose voice it is and where a voicer stands may matter more. We discussed the theoretical and practical implications of these findings in employee voice research.

## 1. Introduction

Employee voice is a key driver of group or organizational success and constructive change [1,2,3]. Given its importance, employees should be able to continuously speak up. However, others’ reactions to voice behavior may not always be positive in the workplace [1,4,5]. Owing to the risks and uncertainty associated with voicing, people may choose not to speak up again [2,6]. However, previous research has not paid sufficient attention to the consequences of voice [3,6,7], and how group members evaluate a voicer remains unsolved.

To enable employees to speak up, we need to understand the social outcomes of voice behavior and the mechanism through which it is produced. Several studies have emphasized that voice increases the status, power, and personal influence of a voicer [4,8]. Consequently, voice can be a potential path to emerging as an informal leader in a group. This study investigates the impact of voice on personal influence within a team, considering that individual influence indicates others’ perceptions of that individual’s status, power, and informal leadership [9].

Furthermore, employee voice is a social phenomenon. It has a target or audience to speak to. Even voice conveying the same content can be recognized or evaluated differently by the supervisor and colleagues in the workplace [1,7,10,11]. Recent research has highlighted the importance of social relationships in the voice process [4,12]. Moreover, most of the previous researchers have focused on Western countries. More recent research has shown that voice behavior in Asian countries is a critical issue for improving the organizations within global competition and fast-changing environments [13,14]. In addition, an employee in Asian countries is influenced by loyalty, hierarchical obligation, and principles of harmony [14]. Therefore, the consequences of employee voice can be more affected by contextual social climates such as leaders or members. Therefore, although there are advances in the empirical and theoretical research on employee voice behavior, this study needs to examine social phenomena by demonstrating how the social relationship affects the ‘voice-influence’ relationship.

In light of this stream of research, we examine the moderating role of leader–member exchange (LMX) and peer relationships in the process of determining the voice outcome. Drawing on social network theory, we especially focus on the separate and joint effects of LMX and peer relationships. Although the main effects of LMX have been well addressed [15,16] in voice research, studies considering LMX as a contextual moderator affecting voice outcomes are still underdeveloped. Therefore, this study investigates how LMX affects the relationship between employee voice and its outcome. Further, another important social contextual factor, peer relationships, still remains understudied. Recent studies have also paid increasing attention to peer reactions to voice [8,10,17]. For example, a central position in the peer relationship was found to be related to the personal influence and voice [18,19]. However, its moderating role in the whole process of voice-influence relationships has not yet been clearly elucidated. Therefore, we need to examine friendship ties among team members, excluding the formal leader, to capture the pure impact of informal peer relationships. These results reveal that LMX and peer relationships affect the voice-influence relationship separately. As such, this study can contribute to developing voice literature.

Moreover, this study goes further to examine the combined effects of these two social relationships. In a real work team, a member’s relationships with the formal leader and with other members (excluding the leader) are inextricably interwoven [20]. Several studies examined the social positions of employees and leaders or the moderating role of average peer-LMX in relation to voice (cf. [18,21]). However, extant studies have not fully addressed the joint effects of the two relationships or discussed their implications. For example, if you develop a high-quality relationship with your leader (high LMX) but occupy a peripheral position in your friendship network (i.e., isolated), your colleagues may interpret your constructive voice as an impression-management act, thereby reducing your influence on the team. In contrast, if a member gets along well with peers and speaks up simultaneously, their voice would be more beneficial in terms of influence within the team. Thus, classifying situations in which particular social relationships enhance or hinder voice behavior and individual influence is an important research topic. Based on the configurational perspective, we investigate this topic by testing a three-way interaction between LMX, peer relationships, and voice.

In conclusion, our research question aims to better understand under what conditions employee voice enhances voicers’ influences in the workplace. Regardless of voice content, voice outcomes may be beneficial or harmful to voice-performers, along with the social context in which they are located. We advance the theory of voice outcomes by examining the possible combination of LMX and informal peer relationships, which in turn delineates the boundary conditions of harvesting voice outcomes for individuals. Thus, we attempt to answer the question of whose voice will be heard, appreciated, or devalued within a team, and understand the factors amplifying or mitigating the impact of voice.

## 2. Theory and Hypotheses

### 2.1. Consequences of Voice Behavior: Individual Influence

Employee voice, referring to the discretionary communication of improvement-oriented suggestions, ideas, options, or concerns, is a critical component in enhancing organizational or work-unit functioning [3,22,23]. The relationship between status and voice behavior has been widely studied [24,25,26]. According to status attainment theory, an employee who engages in voice behavior may enhance his or her status in a group [8,17]. Specifically, overcoming the risks of challenging the status quo and offering a productive suggestion can highlight the voicer’s willingness to benefit the team. As such, voice behavior can encourage team members to make efforts toward accomplishing collective goals [22]. Consequently, an employee who performs voice behavior with communal orientation may be seen as a leader, which leads to an enhanced status for the voicer [8]. Team members also tend to consider a member who speaks up as an informal leader in the team [3]. Therefore, employee voice is positively related to an individual’s influence within a team.

Further, the nonconforming nature of voice behavior can illuminate a voicer’s competence. Nonconformity (e.g., objecting to the status quo and proposing different opinions) directs the focus of the group to the contributions made by the voicer [27]. From the perspective of expectation states theory, this will raise the status of the voicer by fostering the perception of his or her competence. Moreover, demonstrating one’s social skills by actively participating in group communications may lead to higher social positions [28]. As voice behavior indicates an employee’s social skills in terms of verbal expression and the ability to initiate conversations about social concerns, the voicer is more likely to attain a higher status [29]. Based on this reasoning, we propose the following hypothesis.

**Hypothesis** **1. (H1).** *Voice behavior is positively related to individual influence within a team.*

### 2.2. Voice in Social Context: Moderating Role of LMX and Peer Relationships

Voice behavior is target-sensitive, implying that peers’ perspectives of voice and that of the supervisor play a critical role when individuals decide whether to speak up or not [8]. Therefore, it should be noted that voice-influence relationship is located in the midst of various social contextual factors. Prior research implies that a voicer does not acquire the same status because of different social features that affect others’ assessment of the voicer’s behavior [4,8]. Also, depending on the characteristics of the voicer, ideas can be endorsed or not [26]. In other words, because voice behavior is assessed differently based on one’s characteristics, an employee’s social features determine their legitimacy [28,30,31]. In light of the status characteristics theory, status cues depend on the social or demographic groups a voicer belongs to [32]. As social status is conferred by others, it is determined by one’s social relationships [25]. Whereas prior research has deemed demographic characteristics to be socially significant characteristics (e.g., [8]) recent studies have highlighted the importance of social relationships in the voice process [4,12,25]. Furthermore, social contexts, such as the relationship between the sender and receiver of a message, strongly influence the receiver’s cognitive evaluation of persuasion endeavors (e.g., [9,33]). Thus, employees’ social relationships are pivotal factors that affect their voice behavior and influence in a work team. 

### 2.3. Moderating Role of LMX

First, the relationship with the leader can impact the voice-influence relationship. LMX is the quality of relationships between a supervisor and his or her subordinates [34]. Because of time constraints, leaders establish different relationships with each of their workgroup members [35]. Based on characteristics such as mutual trust, respect, and obligation, leaders divide their subordinates into “in-group” and “out-group” [36]. Employees with high LMX can perform better with the support, information, and opportunities provided by leaders [37,38]. Therefore, an employee voice with high LMX may be recognized as potentially beneficial to the work team by the leader and coworkers, which in turn may further improve their influence on the team. 

In addition, employees with high LMX not only receive valuable resources from the leader but also inflated performance ratings because of leniency bias resulting from the leader’s intention to reciprocate [39,40]. Given the challenging nature of speaking up, voice behavior is riskier than other organizational citizenship behaviors [1,23]. However, when employees with high-quality LMX speak up, a leader is likely to assess their suggestions as valuable efforts for the workgroup’s collective interests because of in-group favoritism [41,42]. Further, team members will assess a high-LMX employee’s voice as more legitimate because they yield higher performance than the individuals with low LMX [4,7,38]. As such, when employees with high LMX speak up, their voice tends to be highly evaluated by their leader and coworkers, thereby boosting the voicer’s influence.

**Hypothesis** **2.** **(H2).** *LMX moderates the relationship between voice behavior and individual influence within a team, such that the positive relationship is stronger when a voicer has higher LMX.*

### 2.4. Moderating Role of Peer Relationships

Peer relationships have gained attention as most organizations now pursue a more horizontal structure [43]. With the growing number of work teams, coworker interactions may help an employee’s voice behavior to be welcomed and obtain the favorable consequences of speaking up [8,17]. In social network theory, a focal actor occupying a central position in a friendship network can be regarded as a member with high-quality interactions with other coworkers [18,44]. High centrality in a friendship network allows access to valued resources and emotional support [45,46]. A central member in friendship networks also tends to interact with coworkers sufficiently and thoroughly observe the behaviors of team members while finding an adequate method to represent their thoughts [47]. In this vein, team members support employees who speak up by taking action to address the problem [48]. This is because an employee’s central position in a friendship network makes other team members aware that he or she is valuable in the work team and that the employee’s voice behavior is conducted to accomplish the collective goal [17,18]. Thus, the voice behavior of an employee with high centrality in a friendship network among peers can be perceived as legitimate by other members of the team. Consequently, the voice behavior of the central employee in the friendship network further increases the employee’s influence within a team.

**Hypothesis** **3.** **(H3).** *Peer relationships moderate the relationship between voice behavior and individual influence within a team, such that the positive relationship is stronger when a voicer has high-quality peer relationships (i.e., occupies a more central position in the friendship network with peers).*

### 2.5. Configurational Perspective on Voice and Social Relationships

In this study, we maintain that the two aforementioned social relationships, LMX and peer relationships, have their own interaction effects on individual influence with voice behavior in a team. However, previous social exchange research has suggested that the relationship between the leader and coworkers is inextricably interwoven within a work team [20,49]. In this sense, LMX and peer relationships jointly affect the voice-influence relationship in a complex manner.

Specifically, an employee’s voice may be more strongly associated with their influence when it is combined with both high LMX and quality peer relationships. Employees with high LMX and high centrality in friendship networks can receive specific supports and opportunities necessary for effective job performance through unique relationships with the supervisor [50] and have access to resources from coworkers through collaboration as well [34,51]. Therefore, employees with both high LMX and high centrality rather than those with low LMX and high centrality are likely to receive higher performance expectations, i.e., the generalized anticipation of their competence to contribute to the work. Consequently, they are positively evaluated for their behavior and perceived as more influential in work teams. Furthermore, prior research has found that one critical factor in the persuasive process is the characteristics of the people who speak up, such as credibility, intelligence, and expertise [52,53]. Similarly, if employees are perceived as reliable in a work team, their voice behavior will be evaluated favorably. This leads to a positive impact of the employee’s voice on the team, which strengthens their influence.

Next, although prior research has suggested that employees with high LMX could be evaluated positively by supervisors [41,54], voicers may suffer more from high LMX than low LMX when they have low centrality in peer networks. Even if an employee is regarded as an ingroup member by the leader, he or she would be deemed an outgroup member among peers. People may view the employee’s voice as an impression management or ingratiation, which aims to create desired perceptions of oneself with the application of speaking up to the leader [55]. Team members may begrudge employees with high LMX because they are treated better than others [20,56]. Therefore, the voice behavior of a member with low centrality in peers and high LMX deteriorates individual influence more than that of a member with low LMX.

**Hypothesis** **4.** **(H4).** *LMX and peer relationships jointly moderate the relationship between voice behavior and individual influence.*

**Hypothesis** **4a.** **(H4a).** *With high-quality peer relationships (i.e., occupies a more central position in the friendship network with peers), the relationship between voice behavior and individual influence is stronger for a voicer with high LMX than for a voicer with low LMX.*

**Hypothesis** **4b.** **(H4b).** *With low-quality peer relationships (i.e., occupies a less central position in the friendship network with peers), the relationship between voice behavior and individual influence is weaker for a voicer with high LMX than for a voicer with low LMX.*

**Figure 1:**
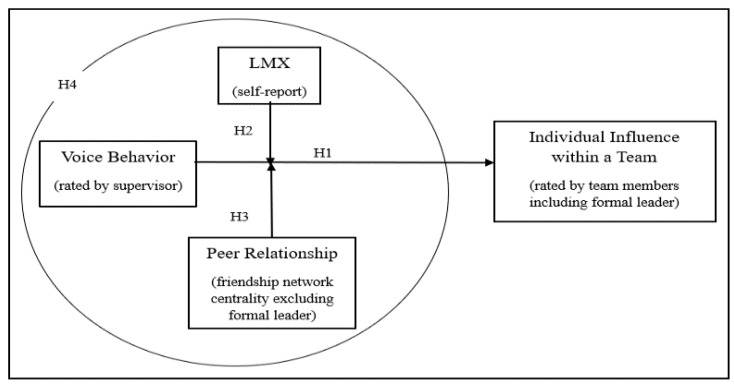
Research Model.

The overall research model is illustrated in Figure 1.

## 3. Method

### 3.1. Sample and Procedure

To test our hypotheses, we collected data from 3 organizations in South Korea. These organizations were an IT company, a manufacturing and retail company, and a public enterprise. Before distributing the survey, we asked HR managers for permission for data collection. We explained this survey’s objective and interviewed a sample of company HR managers. We gained a team roster from organizations with supportive attitudes to the survey. We allocated a serial code to the respondents to recognize their answers. Of the 179 employees in total, 166 replied, representing a response rate of 92.7%. We collected data by questionnaire survey. The survey items and scales of this research were translated into Korean and received precise validity checks using back-translation by a bilingual Korean–American translator independent of our research team [57]. Our survey was approved by appropriate institutional review board (IRB) review. We acquired consents from the respondents and provided them with research outlines, including the purpose of the study, risks and benefits of participation, privacy protection, confidentiality, etc. The questionnaire included measures of employee voice (promotive and prohibitive), LMX, peer relationship, and individual influence. To avoid common method bias, we measured several variables from different sources. Two types of employee voice were rated by the formal leader, LMX was self-reported, peer relationship (centrality in friendship network excluding the formal leader) was calculated using social network analysis software UCINET 8.0, and individual influence was rated by all team members, including the formal leader. After collecting data, we removed unreliable responds and teams with less than 3 members or less than 80% response rates, following previous social network studies (e.g., [58]). Our final sample is 128 respondents of 26 work teams. 

Demographic characteristics of the respondents consisted of male (70%) and female (30%). The age distribution was as follows: 20s (23.4%), 30s (47.7%), 40s (24.2%), and 50s (4.7%). The average organization tenure was 87 months (s.d.= 84.9). The distribution of rank showed that the majority were junior managers (49.2%), followed by staff (25%), senior managers (17.2%), and directors (8.6%). The average team size was 6.82 (s.d: 2.29), with the smallest team comprising 4 members and the largest team containing 10 members. The table with demographic characteristics is given in Table 1.

### 3.2. Measures

#### 3.2.1. Individual Influence

All team members, including formal leader, assessed each member’s influence. We provided the team roster to all team members (respondents). The question was “For each person in your team, on the scale from 1 (none) to 5 (very much), please indicate how much influence the person you checked has in the everyday activities of this team” [45]. Because the formal leader is also one of the team members, we include the formal leader’s rating in the influence matrix. Then, we generated an N × N influence matrix. From this matrix, we calculated the individual’s average influence score rated by all team members.

#### 3.2.2. Employee Voice

We measured promotive and prohibitive voice behavior rated by the formal leader. To date, most of the previous researchers have focused heavily on both promotion and prevention aspects of voice behavior [e.g., [1,3,13]]. Employees can voice in different ways based on promotive or prohibitive focuses. Promotive voices suggest work-related issues that can improve the functioning, while prohibitive voices focus on the current problems or concerns to prevent the current harm. Therefore, we used Liang, Farh, & Farh’s (2012) measure of employee voice behavior. The items of promotive and prohibitive voice are given in Table 2. All scores were on a 5-point Likert-type scale from 1 (never) to 5 (very frequently). Factor analysis revealed that two types of voice items had factor-loadings above 0.60. In our study, Cronbach’s alpha for promotive voice (5-items) was 0.927 and for prohibitive voice (5-items) was 0.893.

#### 3.2.3. Leader–Member Exchange (LMX)

We measured the individual perception of LMX using the seven-item LMX-5 Scale [59]. Items are given in Table 2. The factor loading score of all items was above 0.70. Cronbach’s alpha for 7-items was 0.924.

#### 3.2.4. Peer Relationship

Peer relationship was assessed by team members, excluding the formal leader. We measured indegree centrality in friendship networks among team members using the roster method [60]. Team members were asked to answer the following question: “To what extent did you go out with this person for social activities outside work such as going out to informal lunch, dinner or drinks?” (cf. [58]. The rating was on a 5-point scale from 1 (never) to 5 (very frequently). Then, we create an N × N peer-rated matrix. By using this matrix, we calculated the individual’s valued indegree centrality using UCINET 8.0. Higher centrality indicates that the focal person (member) receives a high level of popularity, trust, and emotional support (i.e., high-quality relationships with peers). 

#### 3.2.5. Control Variables

We controlled for organizational characteristics using firm dummy because the three organizations are different in structure and management style. We also controlled for team size because it influences team processes and outcomes. Demographic characteristics were also included in control variables: age, gender, organizational tenure (in months), and rank.

## 4. Results

Table 3 shows the descriptive statistics and correlations among all variables. Promotive voice (r = 0.430, *p* < 0.01) and prohibitive voice (r = 0.217, *p* < 0.05) were positively related with individual influence. Also, significant positive correlations were between social relationships and individual influence (LMX: r = 0.224, *p* < 0.05; Peer relationship: r = 0.412, *p* < 0.01). 

We tested the hypotheses using hierarchical regression analysis. Table 4 and Table 5 shows the results of regression models. H1 predicted that two types of employee voice (promotive and prohibitive) have a positive effect on individual influence. As shown in model 2 of Table 4 and Table 5, promotive and prohibitive voice was positively related to individual influence (*b* = 0.319, *p* < 0.001; *b* = 0.211, *p* < 0.01). Thus, H1 was supported.

**Figure 2:**
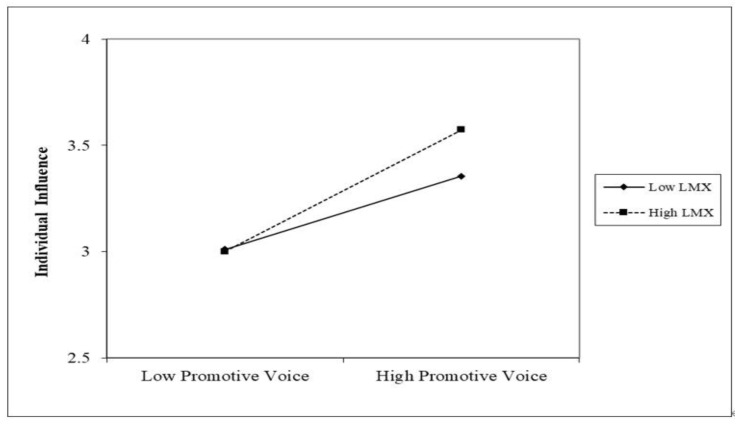
Two-way Interaction of LMX and Promotive Voice.

Next, H2 and H3 proposed the moderating effects of LMX and peer relationship. Following Aiken and West (1991), interaction terms were mean-centered in order to reduce multi-collinearity. As shown in Table 4 (model 4), the interaction of promotive voice and LMX on individual influence was significant (*b* = 0.168, *p* < 0.05), but the interaction with prohibitive voice was not supported (*b* = 0.098, *p* < n.s.). The interaction effect of promotive voice and LMX on individual influence was plotted in Figure 2. It implied that the link between promotive voice and individual influence was stronger when LMX was high. Furthermore, additional tests of a simple slope analysis [61] predicted that the relationship between promotive voice and individual influence was significantly positive when LMX was both high (*b* = 0.420, *p* < 0.001) and low (*b* = 0.252, *p* < 0.01). Also, as shown in Table 4 (model 4), the interactions of promotive voice, prohibitive voice, and peer relationship were not significant (*b* = −0.464, n.s.; *b* = −0.302, n.s.). Thus, H3 was not supported.

H4 was about the three-way interaction of LMX, peer relationship, and voice. To perform a configurational analysis, we entered the three-way interaction term in the final step (promotive voice, f^2^ = 0.739; prohibitive voice, f^2^ = 0.592). As shown in model 5 of Table 4 and Table 5, the three-way interaction of two types of employee voice, LMX, and peer relationship on individual influence was significant. Therefore, H4 was supported for both promotive voice (*b* = 0.889, *p* < 0.05) and prohibitive voice (*b* = 1.096, *p* < 0.05).

**Figure 3:**
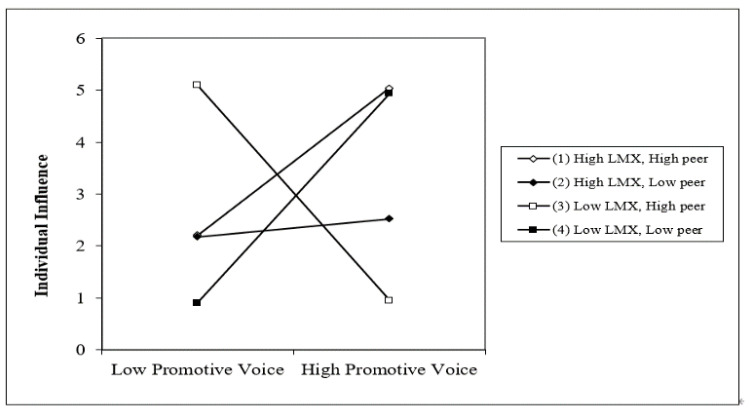
Three-way Interaction of LMX, Peer Relationship, and Promotive Voice.

**Figure 4:**
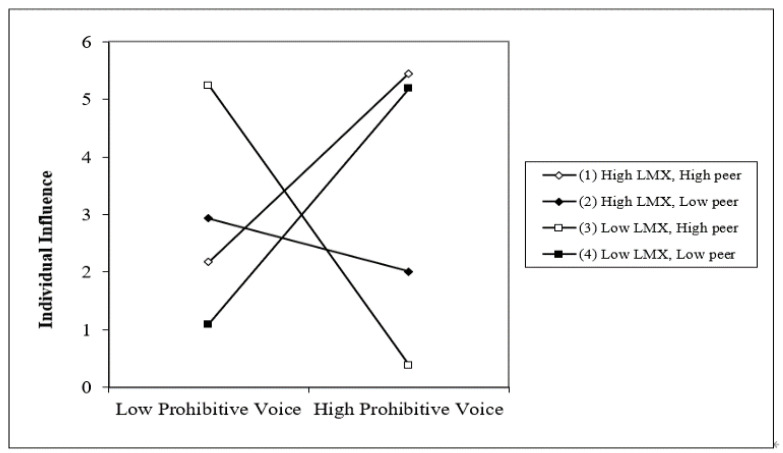
Three-way Interaction of LMX, Peer Relationship, and Prohibitive Voice.

To better understand these results, in Figure 3 and Figure 4, we depicted the interaction effects when the level of centrality in peer networks was high and low (mean±1SD). To interpret the three-way interaction in detail, we employed a simple slope analysis [61]. Figure 3 and Figure 4 showed that the relationship between each type of voice behavior and individual influence was significantly positive when LMX and centrality were both high (promotive voice: *b* = 0.714, *p* < 0.01; prohibitive voice: *b* = 0.820, *p* < 0.05). Conversely, Figure 3 and Figure 4 revealed that voice behavior had a negative relationship with individual influence when a voicer’s centrality was high but LMX was low (promotive voice: *b* = −1.039, *p* < 0.05; prohibitive voice: *b* = −1.215, *p* < 0.05). As for members with high LMX and low centrality, their voices were unrelated to individual influence (promotive voice: *b* = 0.086, n.s.; prohibitive voice: *b* = −0.232, n.s.). Also, employee voice had a marginally significant relationship with individual influence with low LMX and low centrality (promotive voice: *b* = 1.000, *p* < 0.10; prohibitive voice: *b* = 1.022, *p* < 0.10). Following Dawson and Richter (2014), we ran the slope difference test [62]. The results supported the prediction that the relationship between two types of voice and individual influence was significantly stronger when LMX and centrality were both high, and weaker when LMX was low and centrality was high.

## 5. Discussion and Implications

This study aimed to explore the social consequences of employee voice. We found that the two types of voice (promotive and prohibitive) are closely related to the voicers’ influence within a work team. Next, we explored how two different social relationships, LMX and peer relationships, separately and jointly affect the voice-influence relationship. The results showed that LMX strengthened the positive effect of promotive voice behavior on their influence, whereas prohibitive voice behavior did not affect the relationship between voice and influence. These results reconfirm the previous research that promotive voice affects the leader emergence via status, but not prohibitive voice [8]. Compared with promotive voice, prohibitive voice is more likely to invoke potential negative emotions and defensiveness [13]. Even when a voicer has a high-quality relationship with the leader, voice behavior can be viewed as fracturing unity and reduced commitment to collective goals, by which managers may cast doubt on the loyalty of the voicer [1]. In summary, contrary to our expectations, peer relationships did not affect the relationship between employee voice and their influence.

Nevertheless, we still found the combined effects of LMX and peer relationships. When employees with both high LMX and centrality in peer networks speak up, their influence within a team tends to be much higher. However, there is an unexpected effect about the voice outcome. Our results indicate that if employees have high centrality in friendship networks but low LMX, their voice may have adverse effects on their influence. One possible explanation is the nature of friendship networks, such as liking, trust, and closeness (e.g., [59,63]). Centrality in a friendship network implies the extent to which an employee is considered a friend by other coworkers [63,64]. Previous research has suggested that people expect members interacting closely with others to play a role in achieving the socioemotional goals of friendships [20,65]. Thus, a voicer may experience interpersonal conflicts with other members because speaking up involves pointing out the need for improvement of other members and decreases their influence within the team. Our finding further reconfirms that members in collectivistic cultures prefer conflict-avoidance and, therefore, voice can negatively affect their social outcomes [14]. Therefore, this study reconfirms previous research and develops the voice literature by examining the separate and joint effect of LMX and peer relationships on the employee voice and their influence.

### 5.1. Theoretical Implications

This study makes several theoretical contributions. First, this study can answer the recent calls for investigating the social side of voice consequences. Most voice research has focused on the antecedents of voice, such as psychological safety [66], dispositional or attributional factors [67], and leadership behavior [2]. More recently, however, a considerable number of studies began to examine the consequences of voice [4,8,10,68]. Employee voice has a positive influence on the efficiency of organizations but reactions to others’ voice behavior tend to not always be positive [1,4,67,68]. Therefore, to continuously conduct individual voice behavior, the social consequences of voice behavior must be considered. Our results show that employee voice behavior affects individual influence within a team. In doing so, this study contributes to the recent development of voice research from the viewpoint of voice outcomes.

Second, our study contributes to the development of the voice literature from a social relationship perspective. Recent research suggests that not only LMX but also relationships among coworkers impact workplace outcomes [10,50]. Hence, considering LMX and peer relationships as social contextual factors surrounding the voice-outcome relationship will extend our understanding of the interaction of social relationships and voice behavior as well as the direct effect of social relationships on individual influence.

Most importantly, our results pave the way for studying, not only the interactions of the relationships with the leader and peers, but also the optimal balance of LMX and peer relationships from the social network perspective. Although previous social exchange research has argued that LMX and TMX are interwoven in a work team [20], it has not been examined through either theoretical or empirical methods in organizational studies (e.g., [36,69,70]). This study implies that employees can increase their influence by establishing a quality relationship with the leader and their peers at the same time. We also identified the conditions under which social relationships were conducive to individual influence by simultaneously examining LMX and centrality in peer networks. In this sense, focusing only on a supervisor–subordinate relation or coworker social relationships may be misleading voice research. To conclude, we argue that future voice research should address the optimal configuration of social relationships in the process of enhancing positive voice outcomes.

### 5.2. Practical Implications

In a practical sense, our study suggests that managers can consider voice behavior as an important way of increasing members’ influence in the work team. Along with the recent research emphasizing the voice consequences [4,8,71], we also found the positive relationship between voice and individual influences. If coworkers admit an employee is influential, they are more willing to adjust their behavior to assist the influential member. Consequently, employees will recognize that voice behavior is a key success factor in increasing their influence.

More importantly, this study found that voice and influence relationships are affected by various social relationships. Extant studies have claimed that leaders and coworkers provide benefits for employees who speak up, but sometimes leaders and peers may ignore or reject their colleague’s voice [1,10,68]. As employee voice is recognized and processed by targets, the dynamic of voice behavior is not a function of voice expression alone [7]. Thus, the investigation of the combined social relationships such as LMX and peer relations provides a novel avenue for understanding how the relationship between employee voice and individual influence is affected differently. Thus, managers should recognize these combined effects and encourage high-quality social interactions between individuals to maintain employees’ voice behaviors.

In addition, our results imply that LMX is a more critical factor than centrality in friendship networks for employees to gain influence through voice behaviors. Although peer relationships are considered important in the workgroup, their salience may be weaker than that of the vertical relationship between leaders and subordinates. Taken together, this study’s results indicate that managers should consider not only high-quality peer relationships but also sound supervisor-subordinate relationships.

### 5.3. Limitations and Future Research Directions 

Despite its numerous contributions, this study also has several limitations. First, to reduce the common method bias, this study gathered data from two distinct sources: member and leader [72]. However, our cross-sectional research design does not allow us to examine causality among the variables in this study. To overcome these limitations, future research should consider collecting data at multiple time points and test the causality of the relationships between employee voice and their influence within a team more precisely.

Second, we tested our research model using data collected from the firms with collectivistic cultures. The unexpected results of this study may be attributable to specific cultural characteristics. Yum (1988) revealed that the main difference between East Asians and North Americans is their emphasis on communication in relation to social relationships. As our data are from Korean companies which emphasize harmonious social relationships, preserving others’ honor based on relational norms is of great importance, rendering straightforward voice behavior less appropriate [73,74]. This may restrict the generalizability of our findings to other cultural contexts. Therefore, we suggest that scholars consider this topic using a similar design for individualistic cultures in future research.

## 6. Conclusions

As business environments continue to emphasize flexibility and interdependency in workgroups, the importance of voice behavior cannot be overemphasized. This study investigated the consequences of voice behavior by focusing on voice as a social phenomenon. Overall, from a social relationship perspective on voice behavior, this study revealed the effect of voice behavior on employees’ influence in a team. We also shed new light on the importance of the social relationships within a team—both LMX and peer relationships—by demonstrating how different social relationships separately and jointly affect the voice-influence relationship. This study can be a meaningful step toward discovering voice outcomes from the perspective of social relationships. As employees’ proactivity and change-oriented behavior are required more than ever, managing social relationships within a work team will be a critical task for managers to foster employees’ continuous voice behavior in organizations. This study suggests that designing a work team with high-quality LMX and peer relationships may be an effective means of accomplishing goals. Further studies are needed for a deeper understanding of the consequences of voice behavior.

## Figures and Tables

**Table 1:** Demographic Characteristics.

Category		Ratio
Gender	Male	70
	Female	30
Age	20s	23.4
	30s	47.7
	40s	24.2
	50s	4.7
Rank	Junior Manager	49.2
	Staff	25
	Senior Manager	17.2
	Director	8.6

**Table 2:** Reliability and Validity Analysis of Study variables.

Variables		Cronbach’s Alpha	AVE
Promotive Voice	- Proactively develop and make suggestions for issues that may influence the unit. - Proactively suggest new projects which are beneficial to the work unit. - Raise suggestions to improve the unit’s working procedure. - Proactively voice constructive suggestions that help the unit reach its goals. - Make constructive suggestions to improve the unit’s operation.	0.927	0.710
Prohibitive Voice	- Dare to voice opinions on things that might affect efficiency in the work team, even if that would embarrass team members. - Advise other colleagues against undesirable behaviors that would hamper job performance. - Speak up honestly with problems that might cause serious loss to the work unit, even when/though dissenting opinions exist. - Dare to point out problems when they appear in the unit, even if that would hamper relationships with other colleagues. - Proactively report co-ordination problems in the workplace to the management.	0.893	0.775
LMX	- Do you know where you stand with your leader. Do you usually know how satisfied your leader is with what you do? - How well does your leader understand your job problems and needs? - How well does your leader recognize your potential? - Regardless of how much formal authority he/she has built into his/her position, what are the chances that your leader would use his/her power to help you solve problems in your work? - Again, regardless of the amount of formal authority your leader has, what are the chances that he/she would “bail you out” at his/her expense? - I have enough confidence in my leader that I would defend and justify his/her decision if he/she were not present to do so. - How would you characterize your working relationship with your leader?	0.924	0.700

**Table 3:** Means, Standard Deviations, and Correlations.

Variable	Mean	s.d.	1	2	3	4	5	6	7	8	9	10	11
1. Organization 1	0.43	0.50											
2. Organization 2	0.30	0.46	−0.575 **										
3. Team Size	6.82	2.29	−0.008	−0.045									
4. Gender	0.70	0.45	0.025	0.184 *	−0.103								
5. Age	3.73	1.54	−0.455 **	0.647 **	−0.128	0.400 **							
6. Organization Tenure (month)	87.26	84.80	−0.341 **	0.710 **	−0.151	0.312 **	0.797 **						
7. Rank	2.44	0.95	0.067	0.286 **	−0.096	0.444 **	0.589 **	0.499 **					
8. Promotive Voice	3.68	0.68	0.174 *	0.039	−0.109	0.200 *	−0.079	−0.001	0.118	(0.927)			
9. Prohibitive Voice	3.37	0.77	0.228 **	−0.200 *	0.078	0.103	−0.258 **	−0.153	−0.155	0.681 **	(0.893)		
10. LMX	3.62	0.75	0.115	0.093	−0.063	0.139	0.089	0.033	0.192 *	0.245 **	0.064	(0.924)	
11. Peer Relationship	0.66	0.17	−0.138	0.398 **	−0.512 **	−0.019	0.172	0.278 **	0.107	0.121	−0.043	0.031	
12. Individual Influence	3.45	0.58	0.128	0.190 *	−0.261 **	0.111	0.121	0.184 *	0.199 *	0.430 **	0.217 *	0.224 *	0.412 **

Note. n = 128. Gender (female = 0, male = 1), Age (1 = 21–25, 2 = 26–30, 3 = 31–35, 4 = 36–40, 5 = 41–45, 6 = 46–50, 7 = 51–55, 8 = 56–60, 9 = 61+), Rank (1 = non-managerial employee, 2 = middle manager, 3 = first-line supervisor, 4 = senior manager), Organization 1 = IT, Organization 2 = manufacturing and retail). * *p* < 0.05, ** *p* < 0.01, (two-tailed).

**Table 4:** Results of Regression Analyses for Promotive Voice and Individual Influence.

Variables	Model 1	Model 2	Model 3	Model 4	Model 5
Constant	3.524 *** (0.244)	3.483 *** (0.226)	30.214 *** (0.239)	3.187 *** (0.239)	3.194 *** (0.236)
Organization 1	0.360 * (0.137)	0.294 * (0.128)	0.243 ^†^ (0.125)	0.225 ^†^ (0.129)	0.254 * (0.128)
Organization 2	0.472 ** (0.171)	0.335 * (0.161)	0.081 (0.172)	0.058 (0.170)	0.086 (0.168)
Team size	−0.061 ** (0.021)	−0.051 * (0.020)	−0.008 (0.023)	−0.007 (0.023)	−0.010 (0.023)
Gender	−0.006 (0.123)	−0.113 (0.116)	−0.059 (0.113)	−0.019 (0.113)	−0.024 (0.111)
Age	−0.018 (0.065)	0.035 (0.061)	0.054 (0.059)	0.041 (0.059)	0.038 (0.058)
Organizational Tenure	0.000 (0.001)	0.000 (0.001)	0.000 (0.001)	0.000 (0.001)	0.000 (0.001)
Rank	0.053 (0.072)	0.020 (0.067)	0.001 (0.065)	0.013 (0.064)	0.008 (0.063)
Promotive Voice		0.319 *** (0.070)	0.295 *** (0.069)	0.252 *** (0.069)	0.242 ** (0.069)
LMX			0.069 (0.061)	0.106 ^†^ (0.062)	0.112 ^†^ (0.061)
Peer Relationship (PR)			1.128 ** (0.346)	1.141 ** (0.342)	0.896 ** (0.359)
Promotive Voice × LMX				0.168 * (0.080)	0.136 ^†^ (0.081)
Promotive Voice × PR				−0.464 (0.396)	−0.386 (0.393)
LMX × PR				0.696 * (0.340)	1.008 ** (0.370)
Promotive Voice × LMX × PR					0.889 * (0.445)
R^2^	0.182	0.302	0.365	0.405	0.425
Adjusted R^2^	0.134	0.255	0.310	0.337	0.354
F	3.801 **	6.445 ***	6.715 ***	5.958 ***	5.962 ***
∆R^2^	0.182	0.067	0.067	0.031	0.024
∆F	3.801 **	20.602 ***	5.742 **	2.547 ^†^	3.989 *

Note. n = 128. Values represent unstandardized coefficients; Standard errors are noted in parentheses. ^†^ *p* < 0.10, * *p* < 0.05, ** *p* < 0.01, *** *p* < 0.001 (two-tailed).

**Table 5:** Results of Regression Analyses for Prohibitive Voice and Individual Influence.

Variables	Model 1	Model 2	Model 3	Model 6	Model 7
Constant	3.524 *** (0.244)	3.485 *** (0.235)	3.240 *** (0.247)	3.235 *** (0.249)	3.190 *** (0.246)
Organization 1	0.360 * (0.137)	0.321 * (0.133)	0.258 * (0.130)	0.251 ^†^ (0.131)	0.323 * (0.133)
Organization 2	0.472 ** (0.171)	0.495 ** (0.164)	0.216 (0.178)	0.183 (0.180)	0.231 (0.179)
Team size	−0.061 ** (0.021)	−0.067 ** (0.021)	−0.022 (0.024)	−0.022 (0.024)	−0.023 (0.024)
Gender	−0.006 (0.123)	−0.099 (0.121)	−0.046 (0.118)	−0.012 (0.118)	−0.033 (0.117)
Age	−0.018 (0.065)	0.016 (0.063)	0.032 (0.061)	0.029 (0.061)	0.045 (0.061)
Organizational Tenure	0.000 (0.001)	−0.001 (0.001)	0.000 (0.001)	0.000 (0.001)	0.000 (0.001)
Rank	0.053 (0.072)	0.081 (0.069)	0.052 (0.067)	0.050 (0.067)	0.029 (0.067)
Prohibitive Voice		0.211 ** (0.065)	0.185 ** (0.063)	0.179 ** (0.063)	0.157 * (0.063)
LMX			0.102 (0.062)	0.117 ^†^ (0.063)	0.118† (0.062)
Peer Relationship (PR)			1.107 ** (0.360)	1.158 ** (0.360)	0.987 ** (0.364)
Prohibitive Voice × LMX				0.098 (0.087)	0.137 (0.087)
Prohibitive Voice × PR				−0.302 (0.417)	−0.096 (0.423)
LMX × PR				0.676 * (0.326)	1.016 ** (0.360)
Prohibitive Voice × LMX × PR					1.096 * (0.528)
R^2^	0.182	0.249	0.316	0.348	0.372
Adjusted R^2^	0.134	0.198	0.258	0.273	0.294
F	3.801 **	4.930 ***	5.415 ***	4.674 ***	4.775 ***
∆R^2^	0.182	0.067	0.067	0.031	0.024
∆F	3.801 **	10.685 **	5.773 **	1.824	4.311 *

Note. n = 128. Values represent unstandardized coefficients; Standard errors are noted in parentheses. ^†^ *p* < 0.10, * *p* < 0.05, ** *p* < 0.01, *** *p* < 0.001 (two-tailed).

## Data Availability

The data of this study are available from the corresponding author upon reasonable request.
